# Comparison of different predictive models on HFMD based on weather factors in Zibo city, Shandong Province, China

**DOI:** 10.1017/S0950268821002508

**Published:** 2021-12-09

**Authors:** L. L. Liu, Y. C. Hu, C. Qi, Y. C. Zhu, C. Y. Li, L. Wang, F. Cui, X. J. Li

**Affiliations:** 1 Department of Biostatistics, School of Public Health, Cheeloo College of Medicine, Shandong University, Jinan 250012, Shandong, China; 2 Institute of Clinical Trials and Methodology 90 High Holborn London, University College London, London, UK; 3 Zibo Center for Disease Control and Prevention, Zibo 255000, Shandong, China

**Keywords:** Generalised additive model, hand-foot-and-mouth diseases, predict, random forest regression, support vectors regression

## Abstract

The early identification and prediction of hand-foot-and-mouth disease (HFMD) play an important role in the disease prevention and control. However, suitable models are different in regions due to the differences in geography, social economy factors. We collected data associated with daily reported HFMD cases and weather factors of Zibo city in 2010~2019 and used the generalised additive model (GAM) to evaluate the effects of weather factors on HFMD cases. Then, GAM, support vectors regression (SVR) and random forest regression (RFR) models are used to compare predictive results. The annual average incidence was 129.72/100 000 from 2010 to 2019. Its distribution showed a unimodal trend, with incidence increasing from March, peaking from May to September. Our study revealed the nonlinear relationship between temperature, rainfall and relative humidity and HFMD cases and based on the predictive result, the performances of three models constructed ranked in descending order are: SVR > GAM> RFR, and SVR has the smallest prediction errors. These findings provide quantitative evidence for the prediction of HFMD for special high-risk regions and can help public health agencies implement prevention and control measures in advance.

## Background

Hand, foot and mouth disease (HFMD) is a serious infectious disease caused mainly by an enterovirus, threaten mostly children under 5 years old [1–3]. Weather variables, as important predictors of HFMD, which could influence the transmission and reproduction of enterovirus directly or indirectly, have been pointed out in prior study [3–7], but results vary in different regions. For instance, some studies showed that temperature, relative humidity, precipitation, wind speed and sunshine all have an important influence on HFMD cases in Shandong province, Shanghai and Shenzhen [8–10]. However, Huang *et al*. found that wind speed and precipitation have no relationship with HFMD cases in Guangzhou city [11], and Liu *et al*. found that relative humidity has no effect on HFMD cases in Jiangsu Province [12]. In addition, some scholars revealed that temperature, humidity and wind speed have a positive correlation with HFMD, while rainfall and sunshine are negatively correlated with HFMD in studies of Chongqing, Gansu and Shanghai [10, 13, 14]. On the contrary, a study in Hunan province showed a positive effect between rainfall and HFMD incidence, while wind speed has a negative impact on HFMD incidence [15]. The discrepancies of these findings could be likely attributed to the diversities of geographic, socioeconomic and population variables or statistic methods and data sources.

Previous studies attempt to apply various traditional models to predict HFMD incidence, such as SARIMA model, Generalised additive model (GAM), as well as combined models [16]. Currently, the application of machine learning provided new ideas for disease warning measures by applying mathematical models after statistical sorting the historical information of data and applied for the prediction of infectious disease [17–19], such as HFMD, Dengue [20]. However, no studies have proven that machine learning models have been superior to traditional models in prediction, and traditional predictive models are still used. It remains unclear which type models would be better for HFMD epidemic prediction, which hinders HFMD control and prevention [21–23]. In addition, a unified model cannot be found suitable for all regions due to these discrepancies of data source, methods and variables in regions [24]. Therefore, it is urgent to establish a predictive model based on the actual situation of a specific region to predict more accurately.

Zibo city is a high-risk area of HFMD in Shandong Province. The annual average incidence was 127.9/100 000 from 2010 to 2019, exceeding the average level of Shandong Province (93.70/100 000) [17]. Thus, this study aims to explore the optimal model for HFMD prediction based on meteorological factors in Zibo city, helping the formulation and implementation of preventive and control measures of HFMD and providing new objective information for the associated department.

## Materials and methods

### Study area

**Fig. 1.**
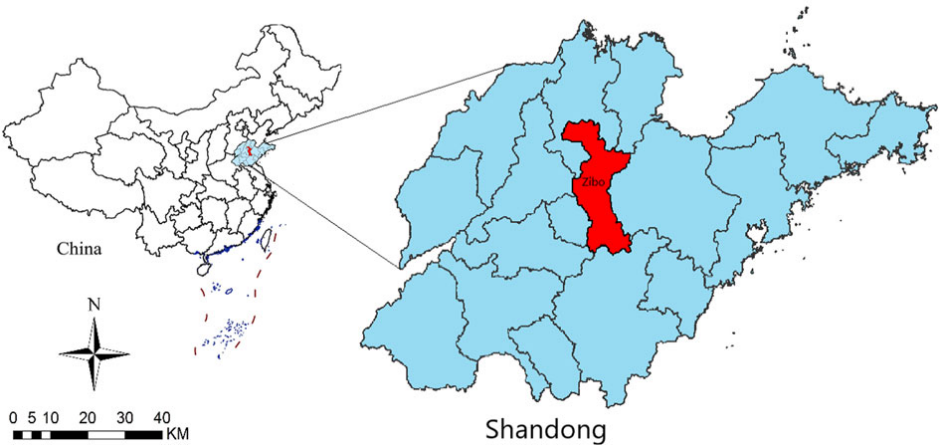
Geolocation of Zibo city in Shandong Province, China.

Zibo is a central city in the Shandong Province of China, located between latitude 35°55′ N and 37°17′ N and longitude 117°32′ E and 118°31′ E (Fig. 1). Zibo city has a temperate monsoon climate. By 2018, the city consists of five districts and three counties, covering an area of 5965 square kilometres, with a permanent resident population of 4.702 million [25].

### Data collection

We collected daily reported HFMD cases during 1 January 2010 and 31 December 2019 from the Diseases Reporting Information System of the Zibo Center for Disease Control and Prevention. Reported cases were divided into two epochs (2010–2018, 2019), the former was defined as a training set to construct models, and the latter as a test set to verify the accuracy of predictive models. Patients were identified according to the diagnostic criteria defined by the National Health Commission of the People's Republic of China (http://www.moh.gov.cn).

Daily climatic data were collected from the Chinese Meteorological Data Sharing System (http://cdc.nmic.cn) and it is publicly accessible, including daily mean, maximum, minimum temperature (°C), precipitation (mm), mean air pressure (k pa), wind speed(m/s), relative humidity (%) and duration of sunshine (h) respectively.

### Model construction

GAM with a Quasi-Poisson family was used to fit the association of weather factors and the number of HFMD [20]. We used categorical indicator variables to control the effects of the weekend and public holidays, and penalised smoothing splines functions to adjust for seasonality and long-term time trends in morbidity [14]. Considering HFMD incubation period range from 3 to 10 days, a lag of up to 14 days was chosen for this study. Each lag day was modelled, and the optimal lag model used to analyse the relationship between meteorological factors and HFMD was selected by comparing the *R*^2^ of models [26]. Akaike's information criterion for quasi-Poisson (QAIC) and previous similar studies were adopted to choose the df for climate factors [7, 27].1
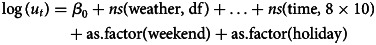
where: *u*_***t***_ is the expected number of HFMD on t day, *β*_0_ is the intercept, *ns*() present penalised smoothing splines.

Support vectors regression (SVR) model maps the nonlinear samples from the original space to the high-dimensional space, where conducts linear regression for finding an appropriate regression hyperplane, that is, the sample fits the optimal regression function, which is used for regression prediction. For the sample (*x*_*i*_, *y*_*i*_), SVR allows a difference closed to *ε* between predicted value *f*(*x*_*i*_) and observed value *y*_*i*_, which is equivalent to building an interval band with a width of 2 *ε*, if the training sample falls into this interval band, it is considered to be predicted correctly [18, 28]. Thus, the SVR problem can be formalised as:2
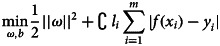
where, 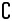 is the constant of regularisation, *l*_*i*_ is the *ε*-insensitive loss function, *ω*, *b* is model parameters to be determined, m is the number of HFMD cases.

Random forest was applied to deal with classification and regression problems. If the CART tree is a regression tree, then the adopted principle is the least mean square deviation [26]. That is, for any partition feature A, the data was divided into sets D_1_ and D_2_ by the partition points, calculating the minimum mean square deviation of D_1_ and D_2_ sets and the corresponding feature and eigenvalue partition points. Random forest regression (RFR) run efficiently on high-dimension data sets, in addition, it is more accurate and robust to noise [19]. The main step of obtaining an optimal model is to determine two parameters: mtry and decision tree (ntree) [29]. When used in regression, mtry is usually 1/3 of all factors in the database. The prediction of CART trees is based on the average of leaf nodes, so the prediction of random forest is the average of the predicted values of all trees. The function is as follows:3
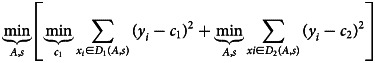
where, *c*_1_ is the sample output mean of *D*_1_ dataset, and *c*_2_ is the sample output mean of *D*_2_ dataset. The predictive validity of the models was evaluated by the criteria of the root mean square error (RMSE), mean absolute error (MAE) and *R*^2^.

Data collection and statistical analyses were performed by excel 2016 and R 4.0.1 software respectively in the study. We used the package ‘e1071’ for support vector regression [30], ‘randomForest’ for the RFR [31] and ‘mgcv’ for GAMs [32] respectively.

## Results

### Descriptive analysis

Daily HFMD counts and weather variables during the study period are shown in Table 1. Daily average cases are 14.26 and the daily maximum count reached 117 cases.

**Table 1.** Summary statistics for daily HFMD cases and weather factors in Zibo city, China, 2010–2019 (*n* = 56 058)

Variables	Mean (s.d.)	Min	P_5_	Median	Max	P_95_
Number of cases	14.26 (17.50)	0.00	0.00	8.00	117.00	52.00
Maximum temperature (°C)	18.74 (10.65)	−8.00	1.00	20.60	38.10	33.10
Minimum temperature(°C)	8.82 (10.58)	−17.70	−8.00	9.80	28.50	23.80
Mean temperature (°C)	13.29 (10.52)	−13.10	−3.60	14.80	32.00	27.52
Relative humidity (%)	6.75 (1.80)	0.00	3.30	6.40	10.00	9.10
Sunshine duration (h)	6.34 (3.74)	0.00	0.00	7.30	13.20	11.50
Precipitation (mm)	981.34 (8.34)	962.00	968.30	918.50	1003.80	994.70
Wind speed (m/s)	1.70 (0.77)	0.00	0.70	1.60	6.90	3.20
Pressure (kpa)	2.0 (8.03)	0.00	0.00	0.00	142.40	11.90

s.d., standard deviation, P_X_, interquartile range.

**Fig. 2.**
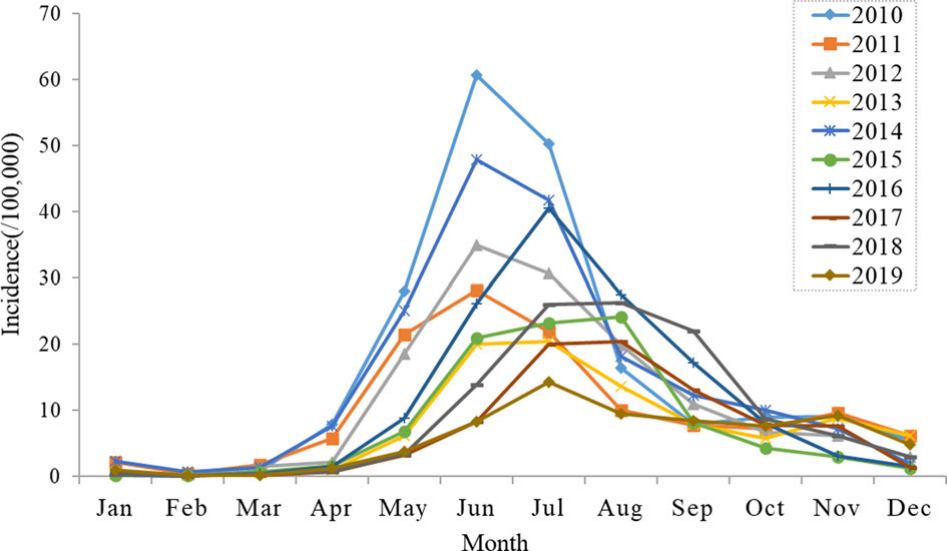
Time series diagram of yearly HFMD cases from 2010 to 2019 in Zibo city, Shandong Province.

**Fig. 3.**
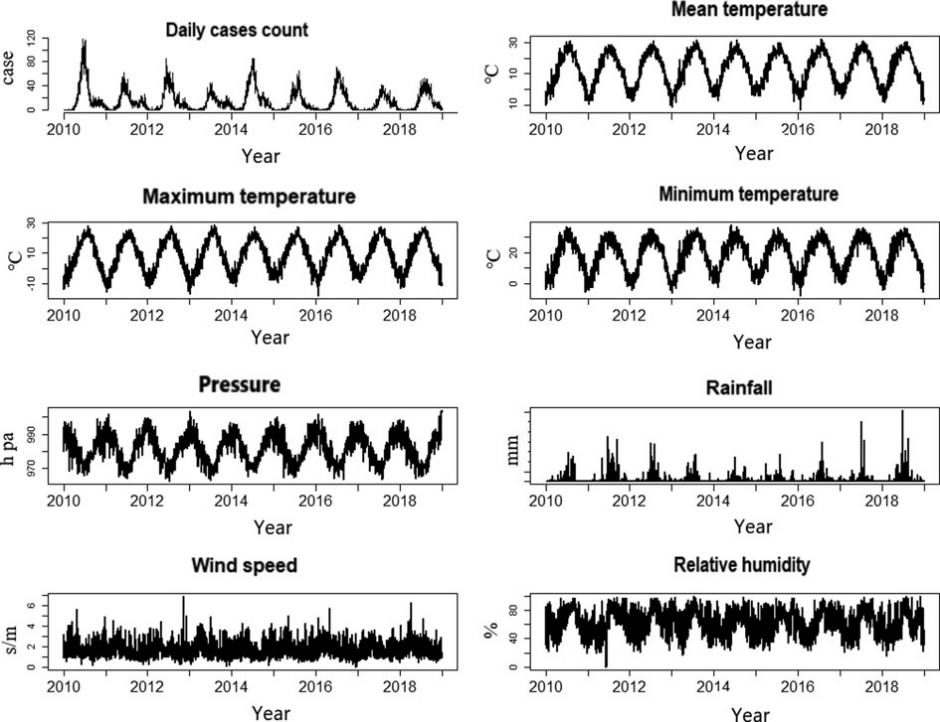
TIme series of yearly weather variables and HFMD cases during 2010–2019 in Zibo city, Shandong Province.

Figure 2 shows monthly HFMD incidence distribution, and Figure 3 displays the time series of daily HFMD cases and weather variables in Zibo city, 2010–2019. We found that the onset of HFMD presents an unimodal trend, increasing from March, concentrating from May to September (Fig. 2), and the onset of HFMD presents obvious seasonality and cycle occur almost every 2–3 years (Fig. 3). In addition, HFMD cases and climate vectors except for wind speed (Fig. 3).

Spearman correlation between weather factors and HFMD cases is depicted in Table 2. Variables positively correlated with the HFMD cases included precipitation, sunshine hours, mean temperature, maximum and minimum temperature and relative humidity, and variables negatively correlated included wind speed and air pressure. While there was no significant correlation between wind speed and sunshine hours and HFMD cases (|*r*| < 0.1). Taking the multicollinearity between variables into account, we can't take the variables that | r | between variables are more than 0.8 into models. From Table 2, we find that there is multi-collinearity between air pressure and the three temperature variables as well as among three temperature variables with each other (|*r*| > 0.8). Based on a previous study, we only take mean temperature into models [33]. In summary, weather variables taken into models are mean temperature, relative humidity and precipitation respectively.

**Table 2.** Spearman's correlation coefficients between meteorological factors and daily HFMD cases in Zibo city, China, 2010–2019

Variables	PRE	PRS	WIN	TEM	RHU	SSD	T_min_	T_max_
PRE	1							
PRS	−0.27	1						
WIN	0^n^	−0.11	1					
TEM	0.19	−0.87	(−0.01) ^n^	1				
RHU	0.52	−0.33	−0.27	0.35	1			
SSD	−0.43	−0.12	0.22	0.20	−0.54	1		
T_min_	0.29	−0.85	(−0.01) ^n^	0.98	0.45	0.06	1	
T_max_	0.11	−0.86	(−0.02) ^n^	0.98	0.27	0.3	0.93	1
Number of cases	0.15	−0.50	(−0.07) ^n^	0.60	0.30	0.09	0.61	0.58

R^n^: No Statistical Significance.

PRE, precipitation. PRS, pressure.

WIN, wind speed. RHU, relative humidity.

SSD, Sunshine duration. TEM, mean temperature.

T_min_, daily minimum temperature. T_max_, daily maximum temperature.

### Comparison of models

The independent variables included in SVR, RFR and GAM are the same, all including average temperature, relative humidity and precipitation respectively.

**Fig. 4.**
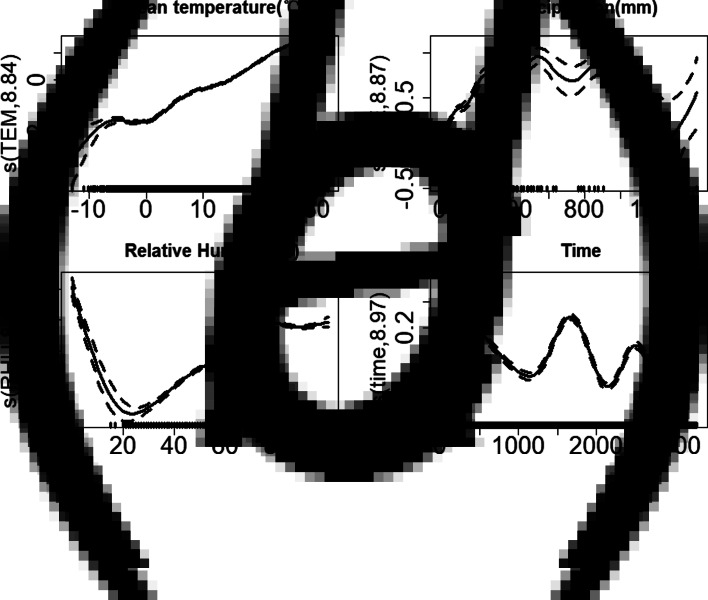
Effects of meteorological factors on HFMD in Zibo city, Shandong Province, 2010–2019(A-D).

Figure 4 (the ordinate represents the smooth fitting value of weather factors to the incidence of HFMD) presents the nonlinear effects of mean temperature, precipitation and relative humidity on HFMD cases. We found the potential risks of HFMD increased as the temperature increased (below the threshold value of 28 °C), then became flatted. Remarkably, the data is relatively sparse and not significant (below −10 °C). As for relative humidity, there is a threshold at 77% about the effect of relative humidity on HFMD cases, when the risk of HFMD increases rapidly (less than 77%) and then decreases slightly and keep stable. While the effect of precipitation on HFMD cases presents ‘M’ shape (when precipitation<600 mm), *s* () increases rapidly, then falls (about 300 mm), then rise quickly (at 400 mm) and more than the previous peak.

**Fig. 5.**
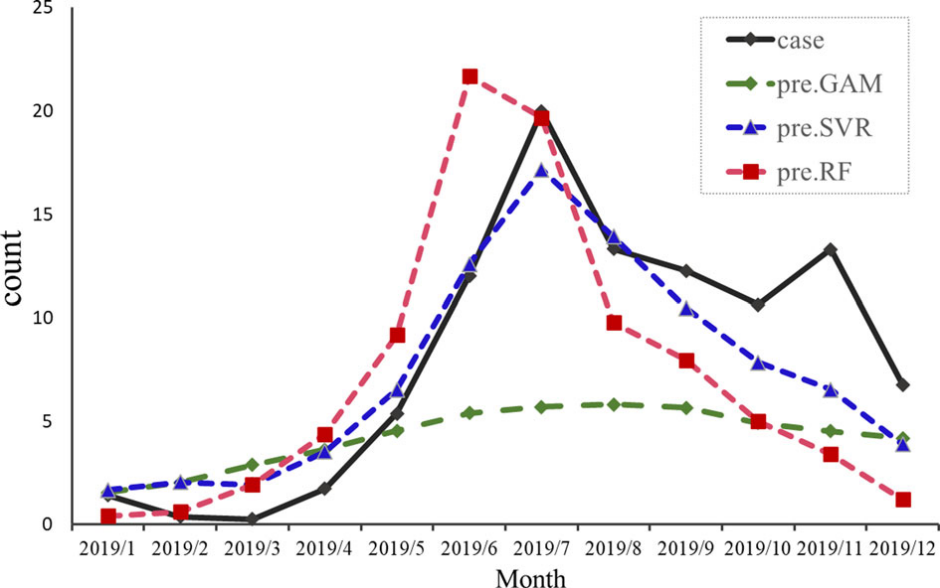
Predictive results of three models in Zibo city, Shandong Province (2019) respectively.

Figure 5 shows the predictive results of the three models for HFMD cases in 2019 in Zibo city. The results of SVR prediction are closer to the observed values, predicting a large epidemic peak from May to August, and have the smallest predictive errors compared to GAM and RFR (Table 3). The accuracy of the three models was ranked in descending order as follow: SVR > GAM > RFR. The prediction results of SVR for HFMD are closest to the observed value, and the trend is roughly coincident to the original trend. By contrast, RFR and GAM can accurately predict the incidence trend of HFMD, but cannot accurately predict the specific number of cases. Therefore, the SVR model was effective and applicable for the prediction of HFMD in Zibo city and RFR and GAM fail to be used to predict HFMD cases.

**Table 3.** Comparison of the predictive performance of three different predictive models

Model	Fitted value (2010–2018)			Predictive value (2019)		
	MAE	MSE	RMSE	MAE	MSE	RMSE
GAM	7.29	125.72	11.21	5.41	55.29	7.43
SVR	9.41	182.37	13.50	1.46	5.44	2.33
RFR	14.83	547.06	23.38	7.99	113.96	10.68

## Discussion

HFMD has been increasingly recognised as a significant health problem, and climate change may increase the burden of HFMD. An early warning could help health agencies to release potential disease outbreaks through the combination of disease surveillance information and climate factors [34]. This study assessed the effect of temperature, precipitation and relative humidity on HFMD cases, and constructed the most appropriate predictive model for HFMD based on meteorological factors.

The purpose of this study is to explore the influence of weather on HFMD and predict the incidence of disease on this basis. Therefore, other factors such as age, immunity of patients and the influence factors of virus infection are not considered, and only meteorological factors are taken into the model. Although the influence of meteorological factors on HFMD has been explored in published studies, the effects of these studies are inconsistent. Effect of temperature on HFMD presents an inverted V-shape in Guangzhou city and Beijing [35]. On the contrary, a linear correlation between temperature and HFMD cases was found in other studies of Guangzhou [34]. Additionally, the impact of wind speed on HFMD has always been controversial. Studies by WangHao *et al.* and Wuxinrui *et al.* found wind speed is a protective factor for HFMD [9, 15], the possible explanation is that wind speed increases the resistance to transmission of viruses, blocking the transmission of viruses, which reduces the chance of infected disease, while some studies show that wind speed is a risk factor for HFMD [10, 13, 14]. In this study, we observed that the effects of temperature, relative humidity and precipitation on HFMD presented nonlinear trends, and there are thresholds, which are consistent with studies in Guangdong province, Japan, Singapore [36–38]. These regional discrepancies could be likely attributed to the diversities of geographic, socioeconomic and population variables or methods and data sources. Although there is no unified conclusion about biological mechanisms of climate factors on HFMD, acceptable explanations in published studies mainly include three aspects: survival and reproduction of pathogens, the host population and environmental factors [39]. Appropriate temperature and humidity are conductive to the propagation of the virus, increasing the probability of disease, and wind speed will accelerate the spread of the virus, thus leading to more infections. Therefore, related departments could use the impact of meteorological factors on HFMD in specific areas to predict the incidence, providing a scientific basis for the prevention and control of HFMD.

Based on the impacts of meteorological factors on HFMD, we constructed and compared three predictive models. The comparison of prediction may help epidemiologists choose the most appropriate model for a given situation. Although three models can solve nonlinear prediction problems, for the predictive efficiency, the order of the three predictive models is SVR > GAM > RFR. GAM excelled flexibly controlling the confounding factors affecting diseases, such as time series, weekend and holiday effect. The adjusted degree of freedom of factors, it has been widely used to predict a variety of diseases, but it is too complex in calculation and sensitive to the choice of parameters. Machine learning methods don't require complex calculations, and the choice of parameters is less likely to cause a large change in error. In contrast, machine learning is less affected by parameter selection. SVR model is a new approach of machine learning provided better performance and greater potential in predicting infectious disease. It uses a nonlinear kernel function followed by linear regression in this feature space and can minimise the actual risk by seeking to minimise the structural risk and better solves problems such as small samples and nonlinearity, which are the most prominent feature of SVR [40]. Because it carries out linear regression in high-dimensional feature space by insensitive loss function and makes use of strong data inclusion to reduce the complexity of the model for a long time. It avoids the traditional process from induction to deduction, achieves the efficient inference from training sample to prediction sample, greatly simplifies the traditional regression problem. Although the SVR model algorithm is simple and has good robustness, it fails to implement for large-scale training sample, which will consume a lot of machine memory and operation time. However, due to fast training speed and two random principles, RFR can process a large amount of data and not easy to generate overfitting problems. In addition, it has a strong ability to make use of data, and obtain the importance of eigenvalue, but takes a long time when the number of decision trees. In summary, for prediction of HFMD in 2019, Zibo city, the results of SVR are closest to the observed value, and has minimal predictive errors.

There are several limitations which should be noted. Firstly, we only consider meteorological factors as risk factors for HFMD, but other influencing factors, such as population density, economic conditions, geographical environment are not taken into consideration, which may decrease the predictive ability of the model [12]. Secondly, due to discrepancy in geographical location, living custom and other aspects, the results may only apply to HFMD in Zibo city, and cannot be extended to other regions with different weather patterns.

In summary, we compared three models based on the influence of weather factors on HFMD incidence, and concluded that the SVR is superior to GAM and RFR, and can be used for forecasting HFMD cases, providing scientific evidence for further application in HFMD prevention and control in Zibo city, Shandong Province.

## Data Availability

The data of this study are available from the Zibo Center for Disease Control and Prevention but restrictions apply to the availability of these data, which are used under licence for the current study, and so cannot be shared publicly.
